# Accepting from the best donor; analysis of long-lifetime donor fluorescent protein pairings to optimise dynamic FLIM-based FRET experiments

**DOI:** 10.1371/journal.pone.0183585

**Published:** 2018-01-02

**Authors:** Kirsty J. Martin, Ewan J. McGhee, Juliana P. Schwarz, Martin Drysdale, Saskia M. Brachmann, Volker Stucke, Owen J. Sansom, Kurt I. Anderson

**Affiliations:** 1 Beatson Institute for Cancer Research, Glasgow, United Kingdom; 2 Novartis Institutes for Biomedical Research, Basel, Switzerland; Cornell University, UNITED STATES

## Abstract

FRET biosensors have proven very useful tools for studying the activation of specific signalling pathways in living cells. Most biosensors designed to date have been predicated on fluorescent protein pairs that were identified by, and for use in, intensity based measurements, however fluorescence lifetime provides a more reliable measurement of FRET. Both the technology and fluorescent proteins available for FRET have moved on dramatically in the last decade. Lifetime imaging systems have become increasingly accessible and user-friendly, and there is an entire field of biology dedicated to refining and adapting different characteristics of existing and novel fluorescent proteins. This growing pool of fluorescent proteins includes the long-lifetime green and cyan fluorescent proteins Clover and mTurquoise2, the red variant mRuby2, and the dark acceptor sREACh. Here, we have tested these donors and acceptors in appropriate combinations against the standard or recommended norms (EGFP and mTFP as donors, mCherry and either Ypet or Venus as acceptors) to determine if they could provide more reliable, reproducible and quantifiable FLIM-FRET data to improve on the dynamic range compared to other donors and breadth of application of biosensor technologies. These tests were performed for comparison on both a wide-field, frequency domain system and a multiphoton, TCSPC time domain FLIM system. Clover proved to be an excellent donor with extended dynamic range in combination with mCherry on both platforms, while mRuby2 showed a high degree of variability and poor FRET efficiencies in all cases. mTFP-Venus was the most consistent cyan-yellow pair between the two FLIM methodologies, but mTurquoise2 has better dynamic range and transfers energy consistently over time to the dark acceptor sRCh. Combination of mTFP-sRCh with Clover-mCherry would allow the simultaneous use of two FLIM-FRET biosensors within one sample by eliminating the crosstalk between the yellow acceptor and green donor emissions.

## Introduction

Fluorescent labels have been used to help solve biological questions for over 5 decades [1, 2]. The development of ‘GFP-biology’ in the early nineties broadened the applicability of such studies, as genetically encoded fluorescent labels could be applied to proteins that were often physically inaccessible to small molecule labelling *in situ* [3]. Detection of photo-physical effects in, and interactions between, such labels has elevated the technique from a purely qualitative imaging process to a potentially quantitative measurement system. Methods such as FRAP and FRET can provide information on molecular dynamics and interactions that were previously unavailable. FRET-based probes have given unique insights into the biochemical activities of a number of critical signalling molecules, such as the dissociation of G-Proteins from GPCRs upon receptor stimulation [4] and the highly localised production of cAMP in cardiac myocytes [5]. Advances in imaging techniques, fluorescent protein biology and DNA manipulation technology have provided researchers with an exciting, but often bewildering, array of options for such experiments.

The fluorescent protein field that began with a single fluorescent protein cloned from the jelly fish *Aequorea victoria* [6] is now crowded with over 100 members, with different combinations of spectral, brightness, photo-stability, lifetime and folding characteristics. Incremental modifications were made to the original GFP to adapt its characteristics for a range of desired experiments, and a number of novel sources of fluorescent proteins (including, but not limited to; the mushroom anemones *Discosoma* [7], the bubble-tip anemone *Entacmaea quadricolor* [8] and the reef building corals *Montipora* [9]) have also been discovered over time. Originally YFP and CFP were developed through mutagenesis of GFP [10], allowing for multispectral imaging. Red variants derived from, or based on, dsRed have been developed and pushed into the far- and infra-red ranges [11]. Photoswitching variants including Dronpa, mApple and Dreiklang [12], and slow folding and maturing ‘timer’ versions like Fast-FT, Slow-FT and Medium-FT [13] have allowed for unique time based experiments, and the pH sensitive pHlourin [14] has been used to highlight specific organelles.

As the range of available protein fluorophores expanded, techniques based upon their interactions also evolved. Förster Resonance Energy Transfer (FRET) is a photo-physical process in which an excited electron within a fluorophore (called the FRET donor) transfers its energy to an electron in a second nearby fluorophore (the FRET acceptor) instead of releasing it as an emitted photon. The excited electron in the acceptor is then able to release the energy as fluorescence. The process has been known to physicists since it was described by Theodor Förster in 1948. It was first demonstrated between a pair of fluorescent proteins *in vitro* in 1996 [15] and it was reported in living cells in 1999 [16]. FRET can only occur between a donor and acceptor whose respective emission and absorbtion spectra overlap (so that the amount of energy transferred is able to excite an acceptor electron) [17, 18]. The greater the overlap between the spectra, the more efficient the energy transfer will be [19] and so a larger the proportion of the donor population undergoes the process. Similarly, FRET is most efficient when the fluorophores are physically close, and this efficiency drops of rapidly (with an inverse 6^th^-power effect) as the distance is increased, meaning it only occurs to a significant degree when the pair are < 10 nm apart [17]. There are two basic approaches to measuring FRET between fluorescent proteins, or any other type of fluorophore; either fluorescence intensity measurements are taken, or a fluorescence lifetime is measured [20].

In the first scenario both the donor and acceptor fluorophores are imaged, and their intensities compared ratiometrically. The presence of FRET within the system can be demonstrated either by bleaching the acceptor, which results in an increased signal from the donor, or by observing changes in the acceptor/donor intensity ratio over time, or in comparison to controls [20]. Such intensity-based ratiometric methods can be performed on a standard fluorescence microscope, provided that the appropriate filters and light sources are available. They are, however, susceptible to artefacts introduced by cross-talk between, and uncontrolled photobleaching events within, the donor and acceptor channels. While attempts can be made to account for this, the variability in fluorescent protein intensity and bleaching responses in cells means that reliable quantification is non-trivial [21].

The second approach measuring FRET is based on the fluorescence lifetime of the donor fluorophore (*i*.*e*. the average time in nanoseconds between absorption and emission of light), which becomes shorter when FRET occurs. FLIM requires more sophisticated instrumentation than intensity-based FRET, but also requires fewer corrections [22], and is more amenable to *in vivo* use [23]. The FLIM approach reduces the risk of acceptor-photobleaching, which can reduce apparent FRET efficiency by rendering acceptors unable to receive energy, because only the donor fluorophore is excited, and is independent of the local concentration variations seen in biological samples [24].

This is particularly important for yellow fluorescent proteins, which are notoriously sensitive to photobleaching and other physical parameters such as pH [25]. Certain donors (such as ECFP) are unsuited to FLIM-FRET, due to their complex lifetime dynamics (*i*.*e*. bi-exponential decay)[26], which has led to the development of new cyan proteins with longer, mono-exponential lifetimes well suited to cyan-yellow FLIM-FRET [18, 27]. Critically, FLIM is also amenable to the use of dark acceptors such as sREACh and its derivatives [28, 29], which presents exciting opportunities to measure two FRET signals simultaneously.

In a modern context FLIM-FRET has its greatest advantages in the realms of *in vivo* measurements [30–32]. Intensity based FRET readouts quickly run into problems as soon as tissue penetration is required, due to the complexities of wavelength-dependent differential light scattering in thick or deep samples [33]. Scattering dramatically affects intensity readings, but does not affect the lifetime distribution of the detected photons. With respect to *in vivo* tissue imaging, longer wavelengths of light have a detection advantage because they exhibit less scattering, but a sufficiently bright short wavelength emitter could comfortably be used for FLIM-FRET as there is no need to be concerned about the scattering differential between donor and acceptor emissions.

In this study we have endeavoured to take as wide a range of considerations as possible into account to identify improved fluorescent pairs for use in FLIM-FRET biosensors, with a particular view to assisting with the development of quantitative biosensor assays that can be consistently applied through *in vitro*, cell-based and *in vivo* experiments. To that end we had three major priorities. Firstly, we wanted to identify pairs of fluorophores that were stable over time under both of our schemes for FLIM detection (frequency domain FLIM and multiphoton-excited multiphoton time domain FLIM). It was also important that they behave similarly across the two platforms. Secondly, we aimed to maximise the potential dynamic range when taking lifetime readings in either the cyan or green channels, by selecting long fluorescence lifetime donors for which the lifetime difference measured between FRET-positive and -negative conditions will be larger, allowing for the detection of smaller changes in FRET efficiency via FLIM. This principle is demonstrated in the Supporting data (S1 Table), in which the expected lifetime measurements of a donor in the absence and presence of a FRET partner accepting energy at an efficiency of 30% are compared. Our third aim was to determine if the sREACh (sRCh) would be a viable acceptor in with our cyan donors, as its nature as a dark acceptor (one that has no visible emissions) could help with the construction of multiplex-biosensor experiments.

With these aims in mind we identified bright, long-lifetime fluorescent protein variants in both the blue (mTurquoise2 [27]–called mTq2 henceforth) and green (Clover [34, 35]–called Clv henceforth) emitting ranges of the spectrum and tested their FRET capabilities relative to the commonly used donors mTFP [18] and EGFP [36, 37] (Table 1). Clv was specifically selected over its relative mNeonGreen as it is reported to have a slightly longer lifetime (3.2 ns for Clv versus 3.0 ns for NeonGreen [35]) and mTq2 for its high quantum yield relative to other Cerulean variants of similar lifetimes, such as mCer3 [27]. Three acceptors were selected for testing with the cyan donors; the ‘classic’ FRET acceptor YPet [25, 38], the improved acceptor Venus (Ven) [39], and the more recently developed dark acceptor sRCh [28, 29]. For the green donors the popular acceptor mCherry (mCh) [40], which has consistently been shown to outperform other RFP variants, and mRuby2 (mR2) [34] were tested. The predicted FRET response of each of these pairs under ideal conditions was calculated and is displayed in Table 2.

**Table 1:** Fluorophores used. Ex = peak excitation wavelength (nm), Em = peak emission wavelength (nm), τ = fluorescence lifetime of donor and QY = quantum yield. Extinction Coefficients (ε) of acceptors are given in 10E3/M/cm.

Donor Fluorophores						Acceptor Fluorophores				
	Ex	Em	τ (ns)	QY	Ref		Ex	Em	ε	Ref
**mTFP1**	462	492	2.8	0.85	[18,19]	**YPet**	517	530	104	[25,38]
**mTq2**	437	474	4.0	0.93	[27]	**Ven**	515	528	92.2	[39]
**EGFP**	489	509	2.4	0.6	[36,37]	**sRch**	510	538	115	[28,29]
**Clv**	505	515	3.2	0.76	[34,35]	**mCh**	587	610	72	[40]
						**mR2**	559	600	113	[34]

**Table 2:** Predicted FRET efficiencies fluorescent protein pairs. The Overlap Integral (J) of the spectra for each pair was calculated using the freeware program **a|e—UV-Vis-IR Spectral Software** (http://www.fluortools.com/software/ae), which uses the normalised emission spectrum of the donor and the normalised excitation spectrum of the acceptor corrected for the published Extinction Coefficient (as detailed in Table 1) to determine the value of J in nm^4^/M.cm. The sources of each spectral dataset are detailed in the Supporting Information. The Forster Radius (r_0_) for the pair was then calculated according to the equation detailed in the Materials and Methods section. Once r_0_ was established an expected FRET Efficiciency at a given separation distance (r) was easily calculated according to the relationship E = r_0_^6^/(r_0_^6^+r^6^).

				Predicted E (%) when:		
Pair	QY_donor_	*J*	r0 (nm)	*r = 5nm*	*r = 6nm*	*r = 7nm*
**Clv-mCh**	0.76	2.50E+15	5.18	55%	29%	14%
**Clv-mR2**	0.76	4.51E+15	5.71	69%	43%	23%
**EGFP-mCh**	0.60	1.94E+15	4.77	43%	20%	9%
**EGFP-mR2**	0.60	3.61E+15	5.29	58%	32%	16%
**mTFP-sRCh**	0.85	3.19E+15	5.50	64%	37%	19%
**mTFP-Ven**	0.85	2.56E+15	5.30	59%	32%	16%
**mTFP-Ypet**	0.85	2.89E+15	5.40	61%	35%	17%
**mTq2-sRCh**	0.93	2.54E+15	5.37	61%	34%	17%
**mTq2-Ven**	0.93	2.04E+15	5.18	55%	29%	14%
**mTq2-YPet**	0.93	2.30E+15	5.28	58%	32%	16%

## Results

### Fluorescence lifetime measurements and FRET efficiencies of fluorophore pairs

In order to compare the fluorescence lifetimes of our selected donors and the relative FRET efficiencies of different donor-acceptor pairings in the context of living cells, fluorescent protein constructs were expressed in HEK 293 cells grown in standard serum-supplemented conditions. These constructs consisted of either the donor alone, expressed without any tags in the cytoplasm of the cells, or a fusion protein consisting of the donor fluorescent protein linked by an 11 amino acid spacer to an acceptor. Fluorescence lifetime readings were taken on the frequency and multiphoton time domain FLIM-systems from separate samples of donor-expressing and donor-acceptor fusion-expressing cells, on three separate occasions per fluorophore pair.

**Fig 1:**
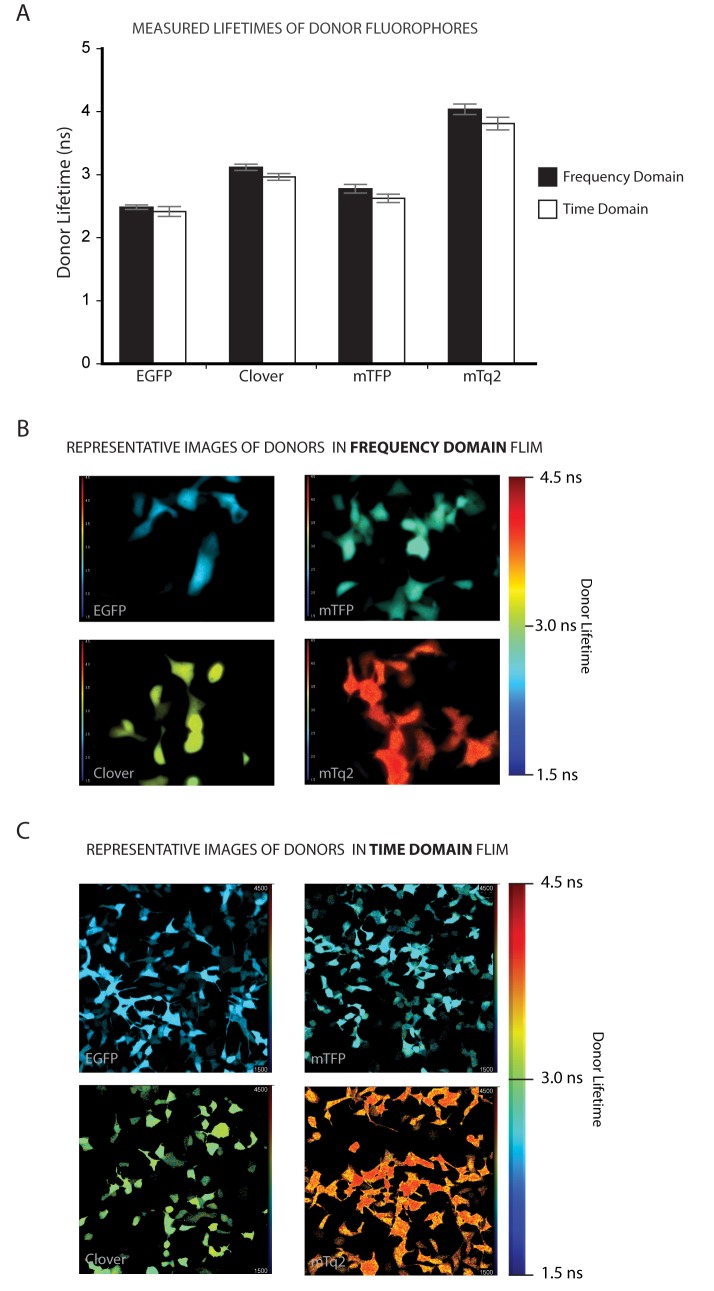
Fluorescence lifetime measurements of FRET donors expressed in mammalian cells. **A.** Fluorescence lifetimes of FRET donors expressed alone in HEK293 cells, measured in both the frequency domain (black bars) and the multiphoton time domain (white bars). Error bars represent the standard deviation in the lifetime measurements of a total 130 > n < 450 individual cells for each bar. **B.** Representative Frequency Domain fluorescence lifetime images for the 4 donors, acquired at 60X magnification on a Nikon TE2000 microscope equipped with the Lambert Instruments Fluorescence Attachment system. Images are false coloured with lifetime information; warm colours represent longer lifetimes and cool colours shorter lifetimes as per the colour scale provided. **C.** Representative Multiphoton Time Domain fluorescence lifetime images for the 4 donors, acquired at 20X magnification on a LaVision BioTec TRIMScope. Images are false coloured with lifetime information; warm colours represent longer lifetimes and cool colours shorter lifetimes as per the colour scale provided.

Fig 1A shows the mean lifetime measurements for the four donors on each system. Under frequency domain detection Clv has a lifetime of 3.12 ± 0.05 ns compared to EGFP’s 2.48 ± 0.04 ns. As expected, the fluorescence lifetime of mTq2 (4.04 ± 0.08 ns) was longer than that of mTFP, which comes in at 2.78 ± 0.07. The multiphoton time domain experiments give similar lifetime differences, with Clv coming in at 2.96 ± 0.05 ns, EGFP at 2.42 ± 0.08 ns, mTq2 at 3.81 ± 0.10 ns and mTFP at 2.62 ± 0.07 ns. Representative images of each donor and pair are shown in Fig 1B. These are largely consistent with published values (Table 1), and it is worth noting that in our hands all four donors exhibit distinct mono-exponential lifetimes (S1 and S2 Figs).

**Fig 2:**
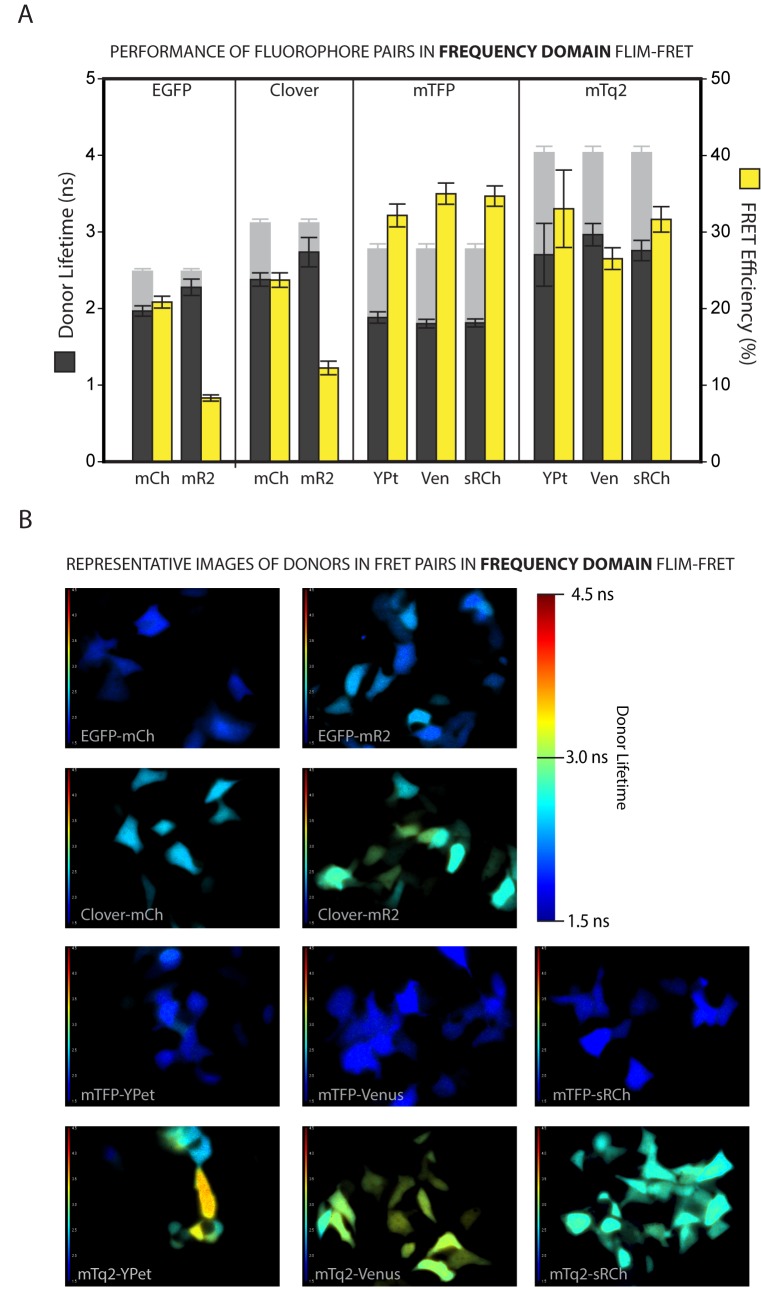
Frequency domain fluorescence lifetime measurements of FRET pairs expressed in mammalian cells. **A.** Frequency domain measurements of fluorescence lifetimes of FRET donors when fused to acceptors and expressed in HEK293 cells (dark grey bars) and the respective FRET efficiencies (yellow bars) calculated from the donor-alone lifetimes from ***Fig 1A*** (shown for reference as greyed-out bars). Error bars represent the standard deviation in the lifetime measurements of a total 90 > n < 155 individual cells for each bar. **B.** Representative Frequency Domain fluorescence lifetime images for the 11 FRET pairs, acquired at 60X magnification on a Nikon TE2000 microscope equipped with the Lambert Instruments Fluorescence Attachment system. Images are false coloured with lifetime information; warm colours represent longer lifetimes and cool colours shorter lifetimes as per the colour scale provided.

**Fig 3:**
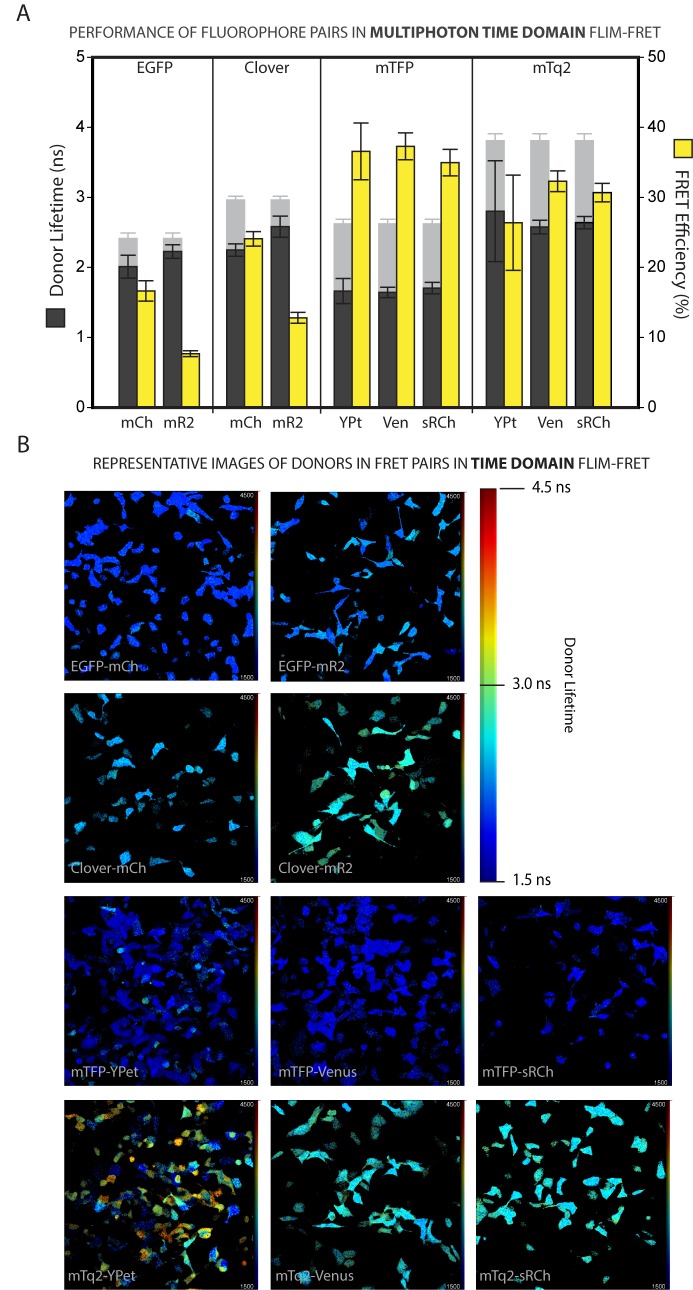
Multiphoton time domain fluorescence lifetime measurements of FRET pairs expressed in mammalian cells. **A.** Multiphoton time domain measurements of fluorescence lifetimes of FRET donors when fused to acceptors and expressed in HEK293 cells (dark grey bars) and the respective FRET efficiencies (yellow bars) calculated from the donor-alone lifetimes from ***Fig 1A*** (shown for reference as greyed-out bars). Error bars represent the standard deviation in the lifetime measurements of a total 105 > n < 145 individual cells for each bar. **B.** Representative Multiphoton Time Domain fluorescence lifetime images for the 11 FRET pairs, acquired at 20X magnification on a LaVision BioTec TRIMScope. Images are false coloured with lifetime information; warm colours represent longer lifetimes and cool colours shorter lifetimes as per the colour scale provided.

Figs 2A and 3A show the mean donor lifetimes measured for each FRET pair (and the resulting FRET efficiencies) for the frequency and time domain systems respectively, as well as representative lifetime images for each pair (Figs 2B and 3B). As a reference point it is worth noting that the EGFP-mCh pairing shows a FD FRET efficiency consistent with a that expected if the fluorophores are separated by a distance of about 6nm (see Table 2), which is about as close as two fluorescent protein barrels can be expected to approach to one another. Indeed, a comparison of all the measured FRET Efficiencies with those predicted at distances of 5, 6 and 7nm shows that for both approaches to FLIM the results most closely resembled those expected from a fluorophore distance of 6nm (S3 Fig). This becomes even more apparent when the mR2 data, which are clear outliers, are eliminated from the analysis, after which the best correlation between predicted and measured FRET Efficiencies is seen when assuming a fluorophore separation distance of 6.0nm (S3 Fig).

The MP-TD result for EGFP-mCh was slightly lower than expected, which may be a consequence of non-linear photophysics resulting from multiphoton excitation. As was predicted, Clv showed a higher FRET efficiency than EGFP when combined with either red acceptor in both of the acquisition systems. It is somewhat surprising, however, thatmCh proved to be a more effective acceptor for both the green donors, with FRET efficiencies of only around 8% for EGFP-mR2 and 12% for Clv-mR2 in both systems. This is in contrast to both our predictions, as detailed in Table 2, and previous intensity based FRET studies of mR2 in which it reported to be an efficient acceptor, with the spectral response of a purified Clover-mRuby2 pair *in vitro* appearing to closely resemble a model based on a 55% FRET Efficiency [34].

In addition to the low measured FRET efficiencies, we see a strikingly high level of cell to cell variability in donor lifetimes within a single dish of cells expressing mR2 as an acceptor, as is evident in the range of colours seen in the false-coloured images (Figs 2B and 3B). Such variation was not seen for either EGFP or Clv alone, suggesting that it resulted from issues of protein maturation and stability on the part of the acceptor itself, or instability in the FRET process between the pair. A simple analysis of the variability in intensity and lifetime data over all the donors and pairs tells us that the degree of lifetime variation does not correlate with that seen in transfection or expression level between cells, for which intensity serves as an approximate readout (S4 Fig). Whatever the source, the variability in our data reveals mR2 as a poor choice of acceptor in mammalian cell based studies.

In the cyan colour range mTq2 showed broadly similar FRET efficiencies to the shorter lifetime donor mTFP, in the region of 30–35%, which is consistent with the predicted FRET efficiencies if the fluorophores are separated by a distance of about 6nm. mTq2 lifetime varied more from acceptor to acceptor, and between systems, than mTFP did. However when combined with sRCh mTq2 performed consistently between the frequency and multiphoton time domain methods with FRET efficiencies of almost 32% and 31% respectively, compared to a predicted 34%. It is worth noting that pairs containing YPet, while providing mean FRET Efficiencies in the range expected at a fluorophore distance of 6nm (Table 2), show similarly high variation in lifetime readouts to mR2, which are particularly emphasised by the long lifetime donor mTq2 (Figs 2B and 3B).

### Defining assay potential of fluorescence lifetime readouts for fluorophore pairs

By converting the change in lifetime reading to FRET Efficiency we enable comparison between donors of different lifetime, but this does not take into account the increased sensitivity that we hoped to achieve with the long-lifetime donors. A more useful comparator for the results is the z-factor (Z’) defined in Eq 1 [41]. This is a parameter commonly applied to high -throughput screens that measures the assay potential of a system, taking into account both the mean (μ) and standard deviation (σ) of the maximum and minimum possible signals of the assay (e.g. fully active versus fully inactive). In our case the maximum lifetime signal is the donor alone and the minimum is the donor lifetime when expressed as a conjugate with each acceptor.

$$z^{\prime }=1-\frac{3(\sigma_{max}+\sigma_{min})}{\left|\mu_{max}-\mu_{min}\right|}$$ Eq 1

**Fig 4:**
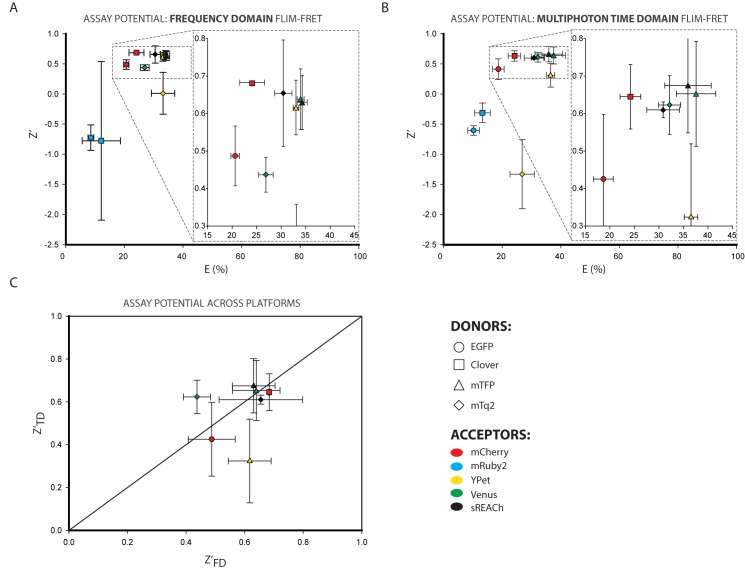
Z-Factor and FRET efficiency as a measure of the single time point assay potential of FRET pairs expressed in mammalian cells. Throughout this figure the donor involved is denoted by the shape of the point (EGFP = circle, Clv = square, mTFP = triangle and mTq2 = diamond) and the acceptor is denoted by the fill-colour of the symbol (mCh = red, mR2 = blue, YPet = yellow, Ven = green and sRCh = black) **A.** Plot of the mean Z’ of the frequency domain lifetime measurements in 3 separate experiments against their mean FRET Efficiency for those experiments. Error bars on both axes represent standard deviation. **B.** Plot of the mean Z’ of the multiphoton time domain lifetime measurements in 3 separate experiments against their mean FRET Efficiency for those experiments. Error bars on both axes represent standard deviation. **C.** Direct comparison of the Z’ values from the two different FLIM systems; pairs that respond similarly on both systems will fall on the x = y diagonal. Error bars represent standard deviation.

This metric provides a read-out of the potential of different FRET pairs and donors of different lifetimes under actual assay conditions. In Fig 4 the Z’ for each pair (mean of 3 repeats of the experiment) is plotted against the mean FRET Efficiency value for that pair; it is immediately apparent from this plot that FRET pairs with similar FRET efficiencies can have substantially different Z’ scores. The closer a Z’ value is to the maximum limit of 1.0, the better the pair. Negative Z’ values represent pairs that give only a small shift in readout from that seen in the donor alone or that have large standard deviations in one or both measurements. The comparison was performed separately for the frequency domain (Fig 4A) and multiphoton time domain (Fig 4B) experiments. The Z’ and E data plotted in these graphs is also summarised in Table 3.

**Table 3:** Assay potential (Z’) and FRET efficiency under frequency and time domain FLIM conditions at t = 0.

	Frequency Domain		Multiphoton Time Domain	
	Z'	% FRET Eff	Z'	% FRET Eff
**Clv-mCh**	0.684 ± 0.003	24.1 ± 2.5	0.645 ± 0.086	24.19 ± 2.12
**Clv-mR2**	-0.778 ± 1.316	12.1 ± 6.5	-0.300 ± 0.163	13.27 ± 2.68
**EGFP-mCh**	0.488 ± 0.080	20.7 ± 0.9	0.425 ± 0.172	18.68 ± 2.00
**EGFP-mR2**	-0.725 ± 0.212	8.5 ± 1.0	-0.592 ± 0.078	10.26 ± 1.99
**mTFP-sRCh**	0.631 ± 0.073	34.4 ± 1.0	0.675 ± 0.127	35.93 ± 4.74
**mTFP-Ven**	0.640 ± 0.081	34.1 ± 0.7	0.653 ± 0.140	37.58 ± 4.01
**mTFP-Ypet**	0.617 ± 0.073	33.1 ± 0.6	0.324 ± 0.195	36.55 ± 1.39
**mTq2-sRCh**	0.655 ± 0.143	30.5 ± 1.8	0.610 ± 0.021	30.86 ± 3.35
**mTq2-Ven**	0.437 ± 0.047	26.9 ± 1.5	0.623 ± 0.078	32.19 ± 2.25
**mTq2-Ypet**	0.011 ± 0.347	33.2 ± 4.0	-1.317 ± 0.573	26.82 ± 4.16

It is clear that Clv-mCh gives a much higher and more reproducible Z’-value than either EGFP pair, along with its higher FRET efficiency, in both systems. mR2 pairs show negative Z’ values as a result of the high standard deviation in the lifetime readings within those datasets.

The cyan-yellow pairs present a more complicated scenario. mTq2-sRCh showed the highest Z’ value when using the frequency domain system, although there was a large degree of variation between the repeats, and it did not have the highest FRET efficiency. That distinction goes to the mTFP-sRCh pairing, with both mTFP-YPet and mTFP-Ven being very close contenders. In fact, in this frequency domain analysis all the mTFP pairs clustered quite closely with high, reproducible Z’ and FRET efficiency scores that result from the small differences in the mTFP lifetime measurements combined with the large lifetime shifts from donor alone to each FRET pair. The behaviour of mTFP as a donor was, however, much less consistent in the multiphoton time domain experiments. mTFP-Ven and mTFP-sRCh displayed the highest multiphoton time domain FRET efficiency and Z’ values respectively, and are closely comparable, but mTFP- high FRET efficiency. The mTq2 pairs mTq2-Ven and mTq2-sRCh have a slightly lower,YPet showed a poor Z’ in spite of a similarly but more reproducible, Z’ than their mTFP counterparts under the multiphoton excitation of the multiphoton time domain system. We would speculate that the differences in fluorescent protein pair performance between platforms most likely result from the different photophysical effects on donor and acceptors that occur when using simple LED illumination versus the high photon-flux, pulsed laser required for multiphoton excitation.

One important consideration for our purposes was the question of how reproducibly the fluorophore pairs behaved between the two systems. This is evaluated in Fig 4C, where Z’_TD_ is plotted against Z’_FD_. An ideal pair that responded exactly the same on both systems would fall on the diagonal y = x; in reality there is some variation between the excitation protocols, so no pair lies directly on that line. However both green-red pairs fall close to the diagonal, while in the cyan-yellow range mTq2-sRCh, mTFP-sRCh and mTFP-Ven all cluster around y = x. By these standards, we can identify Clv-mCh and mTFP-Ven as optimal pairs for use in simple, static lifetime-imaging of multiple samples, but not for repeat measurement of the same sample over time. mTq2-sRCh is also a noteworthy pair due to the increased quantum yield of mTq2 compared to mTFP, which has a number of advantages in live cell and *in vivo* experiments.

### Fluorophore pairs in dynamic studies

**Fig 5:**
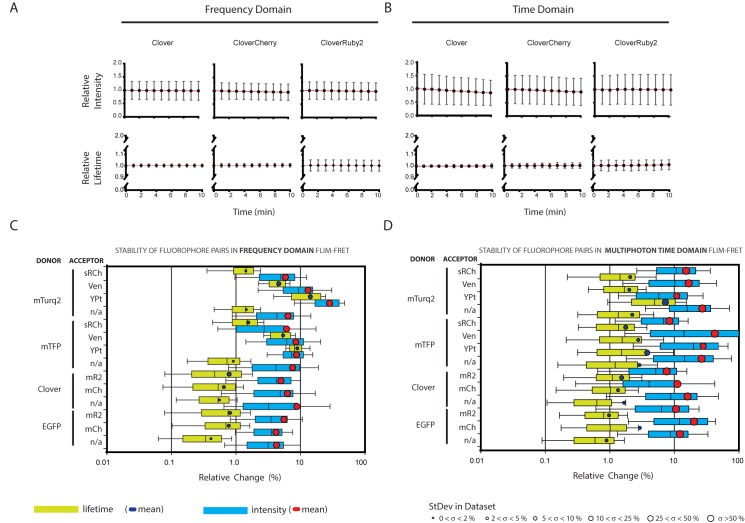
Evaluating stability and variability in fluorescence intensity and fluorescence lifetime. **A.** Example frequency domain time course intensity and lifetime data (in this case, for the green donor Clv). In both cases error bars represent the standard deviation in the normalised intensity and lifetime measurements (each individual cells time course normalised to the mean t = 0 value for that experimental day.) Full data set available in S4 Fig. **B**. Example multiphoton time domain time course intensity and lifetime data (in this case, for the green donor Clv). In both cases error bars represent the standard deviation in the normalised intensity and lifetime measurements (each individual cells time course normalised to the mean t = 0 value for that experimental day) Full data set available in S5C Fig**.** Summary of temporal stability and inter-sample variability in frequency domain data. Box plots represent the distribution of the change (*i*.*e*. the difference between t = 0 and t = 10 values) in each measurement (fluorescence lifetime = lime green box with blue mean symbol; fluorescence intensity = turquoise box with red mean symbol) over the timecourse, showing the range of the 10^th^ to 90th percentiles (whiskers) and the 25^th^ to 75^th^ percentiles (boxes), for all the data sets contributing to the timecourse charts shown in S5 Fig. The median change is marked by an internal black line, the mean change by the centre of the circular points. The size of these circular data points is proportional to the scale of the error in the total data set, with smaller markets indicating a smaller standard deviation in the data. **D.** Summary of temporal stability and inter-sample variability in multiphoton time domain data. Box plots represent the distribution of the change (*i*.*e*. the difference between t = 0 and t = 10 values) in each measurement (fluorescence lifetime = lime green box with blue mean symbol; fluorescence intensity = turquoise box with red mean symbol) over the timecourse, showing the range of the 10^th^ to 90th percentiles (whiskers) and the 25^th^ to 75^th^ percentiles (boxes), for all the data sets contributing to the timecourse charts shown in S6 Fig. The median change is marked by an internal black line, the mean change by the centre of the circular points. The size of these circular data points is proportional to the scale of the error in the total data set, with smaller markets indicating a smaller standard deviation in the data.

Biosensors provide an advantage over standard biochemical assays because of their dynamic nature; they allow repeated measurements over time in the same cells. This is only possible, however, if the photophysics of the interacting fluorophores is stable over time. To test the stability of fluorophore pairs, intensity and lifetime measurements were taken every minute for 10 minutes from cells expressing either a donor alone or a donor-acceptor pair. The collected data are displayed in S5 and S6 Figs: the Frequency Domain results are presented in S5 Fig (average normalised intensity over time) and S5 Fig (average normalised fluorescent lifetime over time), while the Time Domain data are displayed similarly in S6 Fig (intensity) and S6 Fig (lifetime). Representative intensity and lifetime data for Clv, from both frequency domain and multiphoton time domain systems, are presented in Fig 5A and 5B. It is immediately striking that the fluorescence lifetime is far more stable over time (as evidenced by the shallower gradient of a straight line fit through the timecourse data–S2 & S3 Tables), and less variable between samples (as can be seen from the small size of the error bars on the lifetime graphs, which is summarised as an average % error in S2 & S3 Tables), than the intensity measurements. Indeed the data spread for lifetime readings is so tight that in order to distinguish the error bars on some of the lifetime graphs (which represent standard deviation) the y-axis had to be expanded approximately five-fold in comparison to the intensity charts.

Fig 5C and 5D give an overview of the intensity and lifetime stabilities of each donor and fluorophore pair under frequency domain and multiphoton time domain acquisition respectively, by looking at the change in measurement between the start and end of each time course.

It is clear from this data that irrespective of the manner of excitation, fluorescence lifetime is less sensitive to photo-bleaching effects than fluorescence intensity. It is also evident that there is less variation in lifetime measurements between samples than is seen for intensity, with all the intensity points having higher standard deviation (25–50% or > 50%), while lifetime readings largely have standard deviations of less than 10%, with the best pairs having <2% for frequency domain and <5% for multiphoton time domain measurements. The most striking instabilities can be seen easily in the frequency domain graph, where the cyan proteins paired with either YPt or Ven show increased lifetime and intensity change over time and increased variability relative to the donor alone or cyan-sRCh pairs. Similar characteristics can be seen, particularly for YPt in the multiphoton time domain data.

**Fig 6:**
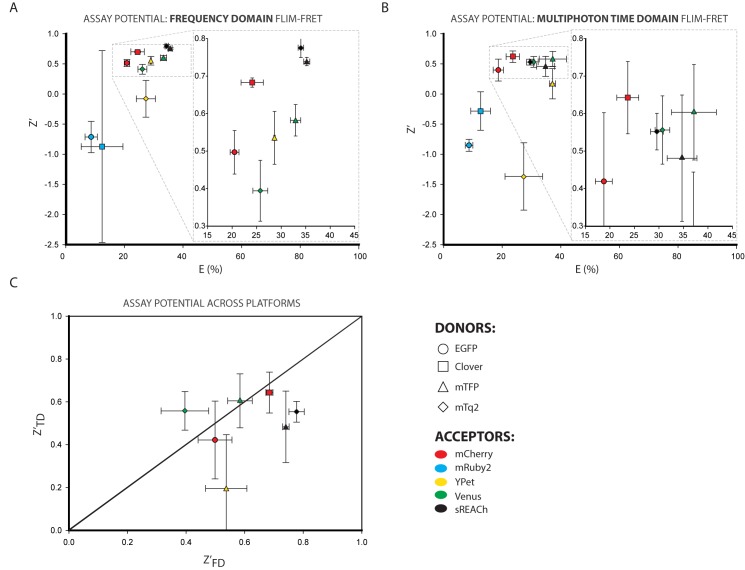
Z-Factor and FRET efficiency as a measure of the dynamic assay potential of FRET pairs expressed in mammalian cells. Throughout this figure the donor involved is denoted by the shape of the point (EGFP = circle, Clv = square, mTFP = triangle and mTq2 = diamond) and the acceptor is denoted by the fill-colour of the symbol (mCh = red, mR2 = blue, YPet = yellow, Ven = green and sRCh = black) **A.** Plot of the mean Z’ of the frequency domain lifetime measurements over a 10 minute time course in 3 separate experiments against their mean FRET Efficiency for those experiments. Error bars on both axes represent standard deviation. **B.** Plot of the mean Z’ of the multiphoton time domain lifetime measurements over a 10 minute time course in 3 separate experiments against their mean FRET Efficiency for those experiments. Error bars on both axes represent standard deviation. **C.** Direct comparison of the Z’ values from the two different FLIM systems; pairs that respond similarly on both systems will fall on the x = y diagonal. Error bars represent standard deviation.

How does this temporal instability affect our evaluation of optimal fluorescent pairs for FLIM experiments? To establish this, the Z’ and E were calculated for all the measurements in each time course, rather than just the first time point as was seen in Fig 4. The Z’ and E data plotted in these graphs are also summarised in Table 4. The frequency domain (Fig 6A) and multiphoton time domain (Fig 6B) results each show the importance of this consideration, as the distribution of the cyan-donor pairs in particular has undergone a substantial rearrangement (relative to the greyed out ‘ghost’ image of the single time point results shown alongside these data in S7 Fig), with all pairs showing drops in Z’ and some reduced Z’ and E values. By plotting the multiphoton time domain Z’ against the frequency domain Z’ (Fig 6C) we can identify the optimal assay potential pairs for use in both single point and dynamic experiments; objectively Clv-mCh in the green-red range and mTFP-Ven in the cyan-yellow range which both fall close to y = x and do not move far between the static and dynamic analysis. However mTq2-sRCh performs better in the frequency domain and similarly in the multiphoton time domain and may be preferable in many situations as a result of its combination of donor brightness and acceptor darkness.

**Table 4:** Assay potential (Z’) and FRET efficiency under frequency and time domain FLIM conditions over 10 minute time course.

	Frequency Domain		Multiphoton Time Domain	
	Z'	% FRET Eff	Z'	% FRET Eff
**Clv-mCh**	0.684 ± 0.012	24.12 ± 2.29	0.642 ± 0.096	23.6 ± 2.3
**Clv-mR2**	-0.888 ± 1.592	12.01 ± 7.12	-0.264 ± 0.321	12.6 ± 3.4
**EGFP-mCh**	0.498 ± 0.058	20.50 ± 0.91	0.420 ± 0.183	18.7 ± 1.7
**EGFP-mR2**	-0.728 ± 0.259	8.28 ± 2.09	-0.835 ± 0.101	8.7 ± 1.3
**mTFP-sRCh**	0.740 ± 0.011	35.28 ± 0.63	0.482 ± 0.168	34.8 ± 3.1
**mTFP-Ven**	0.583 ± 0.042	32.99 ± 1.06	0.604 ± 0.127	37.2 ± 4.6
**mTFP-Ypet**	0.536 ± 0.071	28.68 ± 0.14	0.192 ± 0.252	37.1 ± 1.0
**mTq2-sRCh**	0.777 ± 0.026	34.08 ± 0.57	0.552 ± 0.049	29.6 ± 1.3
**mTq2-Ven**	0.395 ± 0.081	25.78 ± 1.52	0.557 ± 0.091	30.8 ± 1.5
**mTq2-Ypet**	-0.095 ± 0.305	26.98 ± 3.26	-1.354 ± 0.561	27.4 ± 6.4

## Discussion

Our interest in fluorophore pairs for FLIM-FRET was motivated by their application as FRET-biosensors. These engineered proteins can report on the signalling status of specific pathways through a conformational shift that alters the FRET between the two incorporated fluorophores. To date such biosensors have largely been designed with intensity-based FRET methods, rather than FLIM-FRET, in mind. However, FLIM-FRET has a number of advantages over intensity-based methods—more reproducible readings, reduced acceptor photobleaching, independence from intensity fluctuations when working at depth, and the capacity to employ dark acceptors—that can help make biosensor assays more quantitative and more broadly applicable. Modern biological research, especially drug discovery, often requires the use of a pipeline of model systems which span the range from in vitro assays through 2D and 3D cell culture to animal models [42]. To encompass the full range of possible applications for a biosensor, the fluorophores used should ideally perform well on both multiphoton time domain and frequency domain FLIM systems, in spite of their very different illumination and detection methods, as each approach has its advantages in particular types of experiment.

We have used time-domain FLIM in conjunction with multi-photon microscopy to investigate biosensor dynamics in mouse cancer models [43, 44] This is an optimal arrangement when imaging in tissue because MP excitation is good for both TCSPC and increasing the depth of imaging, and TCSPC detection can be performed in wide-field mode, which again improves the depth of imaging. Unfortunately, MP-TCSPC is not the best approach for imaging live cells in culture. Aside from the general limitations associated with laser-scanning compared to wide-field microscopy (e.g.difference in pixel-exposure time), MP excitation produces non-linear photo-bleaching [45] which leads to signal loss and photo-toxicity. Thus in an experimental pipeline in which in vitro imaging precedes in vivo imaging, it is reasonable to expect that these two approaches might be used in conjunction. Furthermore, in the context of scientific reproducibility, it is important to understand the impact of the imaging method on the data obtained, and if possible minimise potential sources of discrepancy.

Time-domain FLIM detection is typically performed using time correlated single photon counting (TCSPC), which requires a pulsed laser source and fast electronics which can determine the time at which a photon is detected from the sample. One major advantage of this technique is that such systems can be equipped with multiphoton lasers to allow the kind of tissue penetration required for *in vivo* imaging. MP-TCSPC does, however, rely on pulsed lasers with a very high photon flux (due to the low probability of multiphoton excitation occuring). This can be damaging to living cells, and introduces the potential for non-linear photo-damage effects [45], which may account for the higher standard deviations in both the FRET Efficiency and Z’ values for the multiphoton time domain data relative to the frequency domain data upon repeated measurement (Fig 4). The confocal laser-scanning approach allows for the highest possible diffraction-limited resolution, but it also introduces limitations to the image acquisition speed.

The most common approach to frequency domain FLIM, on the other hand, is to phase-modulate both the excitation light and the detector sensitivity using epi-fluorescent illumination. This allows for the use of far more gentle LED light sources, and wide-field detection with much shorter exposure times to protect samples That is not to say that such illumination cannot cause photo-damage; the lifetime drift seen in the cyan donors mTFP and mTq2 when paired with either Ypet or Ven on the frequency domain system (S5 Fig) is most likely a consequence of acceptor photo-damage reducing the amount of energy being transferred as the experiment progressed. The image intensifier typically required for such frequency domain lifetime systems, however, also limits the resolution that is achievable, irrespective of the microscope used.

Because we are interested in developing assays based on FRET biosensors, we have chosen to compare FRET pairs using the Z’ value, which is commonly used in assay development, rather than a more conventional estimation of dynamic range based on the signal to noise ratio (S/N). Z’ is an alternative estimation of the dynamic range of an assay and superior to S/N because it incorporates both the standard deviations of the FRET and donor alone measurements, as well as the difference between their means [Zhang et al., 1999]. As a general rule, Z’ values greater than 0.5 indicate a good assay. Therefore we would suggest that any chimera having a Z’ of less than 0.5 is not a good starting point for assay development based on a FRET biosensor. Unlike the chimera, in which the separation between donor and acceptor is easily determined by the linker length, biosensor geometries are more variable and therefore likely to have lower Z’ values.

In the crowded fluorescent protein market there were a few candidates that stood out as having potential for cross-platform FLIM-FRET experiments. The cyan fluorescent mTq2 was designed to have a maximised brightness (with both a high quantum yield and a high extinction coefficient), which could help overcome the issues of photo-damage resulting from pulsed laser excitation by reducing the exposure time, while also having the longest recorded lifetime of any fluorescent protein (4.0 ns). Long-lifetime fluorescent proteins are perfect as FLIM-FRET donors because, compared to their shorter-lifetime counterparts, the same amount of energy transfer will result in a bigger change in measured lifetime, which is easier to reliably detect and provides a larger dynamic range (seen in our data as the larger nanosecond difference between donor-alone and FRET-pair lifetimes–Figs 2 and 3). The same considerations of brightness and fluorescence lifetime made Clv an attractive option as a green donor, with its high quantum yield and lifetime of 3.2 ns. FRET donation from Clv is significantly better than from EGFP, and while the measured FRET efficiency of mTq2 pairs is generally a little less than we see for mTFP the Z’ analysis we have used in Fig 4 demonstrates that mTq2.is a more effective pairing with the dark acceptor sRCh, in which the maximum and minimum lifetime values can be better distinguished over repeated measurements. In our hands, both these donors of interest also have mon-exponential lifetime decays (S1 and S2 Figs).; this is important because it simplifies the fitting of data, and opens up the possibility of trying to fit biosensor data with a bi-exponential function to identify proportions of active and inactive probe molecules. The introduction of a FRET scenario with different on and off states to a system in which the donor already has a bi-exponential lifetime becomes far more complex, and therefore more difficult to interpret. In the acceptor arena, sRCh stood out as a protein of interest due to its dark nature. sRCh has, remarkably, just two amino acids different from its precursor YFP (the specific mutations are Y145W and H148V) but this changes its characteristics dramatically; it still absorbs as efficiently as the parental YFP, but no longer emits any photons. Such an acceptor would be worse than useless for an intensity based FRET approach, but is perfect for FLIM, an approach in which only the donor lifetime is measured. This elimination of acceptor emission from a cyan-donor system leaves the green-through-yellow region of the spectrum (the 550 to 600 nm range) free to be used for an additional probe, either a second FLIM-FRET component or a marker signal that provides context to the FLIM-FRET measurement. This dark acceptor proved to be the most stable of the tested acceptors for both cyan donors, with both mTq2-sRCh and mTFP-sRCh showing the lowest variability in lifetime measured over time in the frequency domain experiments (Fig 5C).

We took the time to analyse the behaviour of the fluorophore pairs over a time course, rather than just in single time point experiments, because in order to use the pairs in dynamic biosensor assays we must be able to rely on consistent fluorophore behaviour. This has demonstrated that some probes (for example any using YPet as an acceptor) could be subject to signal changes over time based on the instability of the pair; this artefact has the potential to mask small changes in activities that may have real biological consequences. As a result it is our strong recommendation that in any dynamic probe experiment control experiments looking at probe behaviour over time must be performed to ensure any changes seen in the experimental data are biological, not photophysical, effects. Particular care should be taken when working in organelles of dramatically different chemical composition to the cytoplasm, as the effect of pH on fluorescent lifetime may vary in degree from on fluorescent protein to another.

**Fig 7:**
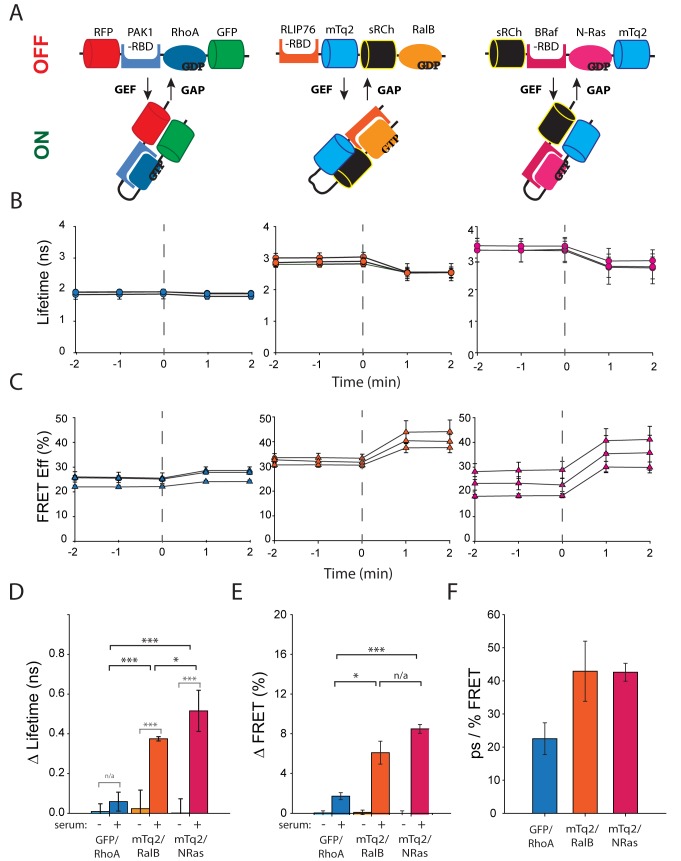
Comparison of an existing GFP-RFP GTPase Biosensor with novel mTq2-sRCh GTPase biosensors. **A.** Schematic representations of the GTPase biosensors tested: a GFP-RFP based RhoA-Raichu [43] (left); a new mTq2-sRCH based NRas-Raichu (right); and a modified version of an alternative geometry RalB probe [46] (centre). **B.** Fluorescensce lifetime responses of serum starved HEK 293 cells transiently expressing GTPase biosensors to 20% serum stimulation at t = 0. Measurements were taken 1 and 2 minutes before and after the central (t = 0) image at which the stimulus added. Each plot represents one repeat of the experiment consisting of the mean lifetimes from 10 < n > 30 cells spread over at least 3 dishes. Error bars represent standard deviations. As above, the GFP-RhoA data is in the left panel, the mTq2-RalB data in the centre and the mTq2-NRas on the right. **C.** FRET Efficiency of the time course data seen in 7B, in which each time point for each day was normalised to the equivalent time point of the average of the donor alone control time courses that underwent the same stimulation protocol on the same day. Error bars represent the percentage error calculated from the standard deviations in the FRET-lifetimes. As above, the GFP-RhoA data is in the left panel, the mTq2-RalB data in the centre and the mTq2-NRas on the right. **D.** Average difference in fluorescence lifetime before and after stimulation compared to the t = 0 value of each time course. Error bars represent standard deviations, and significance was determined using a Rank Sum Test. Statistically significant differences are marked with asterisks. One asterisk represents 0.05 < p > 0.01, two asterisks 0.01 < p > 0.005 and three asterisks p < 0.005. **E.** Average difference in FRET Efficiency before and after stimulation compared to the t = 0 value of each time course. Error bars represent standard deviations, and significance was determined using a Rank Sum Test. Statistically significant differences are marked with asterisks. One asterisk represents 0.05 < p > 0.01, two asterisks 0.01 < p > 0.005 and three asterisks p < 0.005. **F.** Sensitivity of biosensors in terms of picosecond lifetime change per percentage unit of FRET Efficiency after stimulation. Error bars represent standard deviations.

As proof of principle for the effectiveness of these qualities (long donor lifetime, excellent FRET efficiency and Z’ values, and dark acceptor) we demonstrate in Fig 7 that GTPase biosensors using the FRET pair mTq2-sRCh show greater sensitivity than the existing GFP-RFP format that we have worked with. The design of these three probes is shown schematically in Fig 7A. Upon acute stimulation of serum starved cells (20% serum stimulation) our two mTq2-sRCh probes, of varying geometry, show far more distinct changes in measured lifetime (Fig 7B) and consequently in FRET Efficiency (Fig 7C) than the GFP-RFP construct. A more detailed analysis of these data confirm that the change in lifetime between unstimulated and stimulated time points is not statistically significant for the GFP-RFP based biosensor while both mTq2-sRCh containing constructs provide statistically significant changes (Fig 7D). Similarly we can see that the change in lifetime measured from stimulated cells is significantly higher for both mTq2-sRCh based sensors. The significance of this difference is maintained when the data is transformed into FRET Efficiency (Fig 7E), which takes into account the different fluorescence lifetimes of the two donors. Thus we can show that mTq2-donor biosensors are more sensitive than the GFP-donor biosensor, displaying a lifetime change of about 40ps per % FRET Efficiency compared to the GFP baseline of approximately 20ps/ per % FRET. This validation of the mTq2-sRCh pairing for FLIM-FRET biosensors brings complex, context informed FLIM-FRET experiments a significant step closer.

### Conclusions

The data presented in this study show that when designing a FLIM-FRET experiment involving either simple colocalisation experiment or more complex biosensor assays, the selection of appropriate fluorophores is critical to success. We have found that in the green-donor category the combination of Clv and mCh performed well in dynamic experiments across differing FLIM platforms, and provided a better dynamic range than EGFP. In the cyan-donor category mTFP-Ven demonstrated the best reproducibility between multiphoton time domain and frequency domain platforms. However, the cyan donor mTq2 had the best dynamic range and we would recommend combining it with the dark acceptor sRCh to achieve reliable dynamic data. Critically the mTq2-sRCh pairing can be used in combination with Clv-mCh for double-biosensor experiments. As the fluorescent protein field continues to expand new options will no doubt become available; when they do considerations of lifetime stability, pair compatibility and reproducibility within and between platforms should be carefully evaluated in order to get the most from FLIM-FRET experiments.

## Materials and methods

### Molecular biology

The mammalian expression vectors used for single donor fluorescent proteins are summarised in S4 Table. Donor-acceptor fusion proteins were based upon the Clontech pEGFP-C2 vector, which was cut at the HindIII and XbaI sites. The plasmid resulting from the insertion of a HindII/XbaI flanked mTq2 sequence into this site (creating a 21 amino acid spacer between the fluorescent proteins) was the digested with BamHI and BglII and religated to shorten the linker. The BamHI site was reintroduced, while also correcting the frameshift effect of the blunting and religation, using site directed mutagenesis (QuickChange Lightning, Agilent). This resulted in a vector with an 11 amino acid spacer (SGRTQDPPVAT) between two fluorescent proteins. Donor and acceptor fluorescent sequences were switched using standard molecular cloning techniques, with donors inserted between the NheI/BamHI sites and acceptors between the BamHI/XbaI sites. The primers, designed to maintain the 11amino acid linker between constructs, and the template plasmids used, are detailed in S5 Table. The GFP-RFP-RhoA biosensor was previously described [43], and the mTq2-sRCh-NRas biosensor was derived from this, using using standard molecular biology techniques to switch the fluorophore for those detailed above and BRaf-RBD and N-Ras sequences synthesised to order by Genewiz. The mTq2-sRCh-RalB biosensor was a modified form of that previously published by Der *et al* [46], with the fluorophores swapped out using standard molecular biology techniques and the Sec5 binding domain replaced by the RLIP76 (Ralbp1) binding domain sequence, also synthesised to order by Genewiz.*Cell Culture and Transfection*

FRET donor proteins, FRET-pair fusions, and biosensors were expressed in HEK293 cells cultured as standard in DMEM supplemented with 10% foetal bovine serum, 2mM glutamine, 100U/mL penicillin and 100ug/mL streptomycin. Cells were transfected using Lipofectamine-2000 (Invitrogen), according to manufacturer instructions. Cells to be stimulated were serum starved over night.

### Frequency domain fluorescence lifetime imaging

Frequency domain lifetime images were acquired at 60X magnification on a Nikon TE2000 microscope equipped with the Lambert Instruments Fluorescence Attachment system, modulating the excitation light and the sensitivity of the intensifier at a frequency of 40MHz. The system functions with a lifetime resolution of <100ps, according to the manufacturer’s specifications. LEDs emitting at wavelengths of 445nm and 491nm were used to excite cyan and green fluorescent proteins respectively. Reference measurements where made from fluorophores of known fluorescent lifetimes; fluorescein (τ = 4.000 ns) for 445nm excitation and erythrosinB (τ = 0.086 ns) for 491nm excitation. For time courses the software was set to take a FLIM image every minute for 10 minutes and the Nikon Perfect Focus System was used to prevent Z-drift. Lifetime information from individual cells was extracted using the tools available in the accompanying LI-FLIM software.

### Multiphoton time domain fluorescence lifetime imaging

Multiphoton time domain lifetime images were acquired at 20X magnification on a LaVision BioTec TRIMScope, which functions with a temporal bin size of 80ps, according to the manufacturer’s specifications. Time courses were performed by the operator initiating a scan every minute for 10 minutes. Time courses in which refocussing was required were discounted. The TCSPC data files were exported from the La Vision ImSpector Software and lifetime information from individual cells was extracted by fitting the decays from selected areas in the FLIMfit software tool (version 4.7.3) developed at Imperial College London, which has more sophisticated fitting protocols and allows for the fitting of bi-exponential models should it be necessary.

### Calculation of expected FRET efficiencies

The Overlap Integral (J) of the donor emission and acceptor excitation spectra for each pair was calculated using the freeware program *a|e—UV-Vis-IR Spectral Software 1*.*2* (http://www.fluortools.com/software/ae). The sources of each spectral dataset are detailed in the Supporting Information. The Forster Radius (r0) for the pair was then calculated according to the equation below [47], in which we follow the convention of assuming the interdipole orientation factor, κ^2^, to be 2/3[47] and using n = 1.4 as the refractive index of cells in culture [48].

$$r_{0}=0.02108{\left(\kappa^{2}\phi_{D}n^{-4}J\right)}^{1/6}$$

The other parameters in the equation are ϕ_D_, the quantum yield of the donor, and the overlap integral, J, as calculated above. This equation gives r_0_ for the pair–the separation distance at which 50% FRET would be expected between the fluorophores. Expected FRET efficiencies can then be calculated for any given separation distance r (in nm) using the following equation [47]:

$$E=\frac{{r_{0}}^{6}}{{r_{0}}^{6}+r^{6}}$$

### Data handling

Lifetime measurements were made from between 10 and 20 cells per dish, with no more than 3 repeats being performed per day up to a total of 9 dishes per fluorophore pair. All FRET efficiencies were calculated relative to donor alone measurements made in the same cell type, on the same day. For Z-factor calculations the mean and standard deviation of the lifetimes measured for a pair and its donor alone control in one day were taken, with each day of experimentation considered one repeat (n = 3). The complete data set for this study may be found on the Biostudies Website (https://www.ebi.ac.uk/biostudies/) under the accession number S-BSST84.

https://www.ebi.ac.uk/biostudies/studies/S-BSST84

## Supporting information

S1 Fig Representative phasor plots of donor frequency domain data confirm single fluorescence lifetimes of donors. Phasor plots are a convenient representation of frequency domain lifetime data, with each point representing a vector from the origin, the length of which represents the modulation shift between the excitation and emission waves, at an angle ϕ from the X-axis which equals the phase shift between the excitation and emission signals. As a consequence the point [1.0] represents 0ns lifetime and the origin an infinite lifetime. Critically, data from a fluorophore that has a mono-exponential decay cluster on the unit circle, as can be seen to be the case for all 4 donors used in this study.
(EPS)

S2 Fig Representative decay curves for donor time domain data confirm single fluorescence lifetimes of donors. The grey circles represent the recorded data. The solid black lines show a single exponential fit to the data, the coloured dashed lines a double exponential fit. In all cases the two-component equation does not improve the quality of the fit over the single exponential, and can only be applied to the curve by assuming a negative lifetime contribution from the shorter component, which is physically impossible.
(EPS)

S3 Fig Comparing predicted and measured FRET efficiencies. **A.** Predicted FRET Efficiencies at fluorophore separation distances 5nm (*orange circles*), 6nm (*purple squares*) and 7nm (*green squares*) (as reported in ***Table 2***) plotted against the Frequency Domain (left panel) and Time Domain (right panel) data. Straight line fits through the origin show that the results most closely resemble those expected at a 6nm separation (as this fit has the gradient closest to 1). Solid black lines represent the fitted line and the dotted lines the 95% confidence intervals of the fit on each side. The two points that fall outwith this interval are EGFP-mR2 and Clv-mR2 in both cases. **B.** Mean measured FRET Efficiencies for pairs with mCh, YPt, Ven and sRCh as acceptors plotted against Predicted FRET Efficiencies at separation distances of 5.9 nm (left panel), 6 nm (middle panel), and 6.1 nm (right panel). When the mR2 data–which are clear outliers that do not perform as predicted–are removed, the data very closely resemble that expected from a 6 nm separation distance between fluorophores, with a gradient of 0.96 and R^2^ value of 0.9928. Solid black lines represent the fitted line and the dotted lines the 95% confidence intervals of the fit on each side.
(EPS)

S4 Fig Testing for a relationship between lifetime variability and variations in intensity. Over the two distinct data sets (Frequency Domain measurements, in orange, and Time Domain measurements, in blue) there is no significant correlation between % StDev in lifetime and % StDev Intensity, as in both cases the Spearman Correlation P-Value is > 0.05. Correlation Coefficients and P-Values were calculated using Sigma Plot (Version 11).
(EPS)

S5 Fig Collected time course data for frequency domain fluorescent lifetime images. **A.** Frequency domain time course intensity data for all donors and donor-acceptor pairs. Error bars represent the standard deviation in the normalised intensity and lifetime measurements (each individual cells time course normalised to the mean t = 0 value for that experimental day.) 90 > n < 155 for FRET Pairs and 130 < n < 450 for donor alone samples (as separate donor measurements had to be made for every experimental day). **B.** Frequency domain time course lifetime data for all donors and donor-acceptor pairs. Error bars represent the standard deviation in the normalised intensity and lifetime measurements (each individual cells time course normalised to the mean t = 0 value for that experimental day.) 90 > n < 155 for FRET Pairs and 130 < n < 450 for donor alone samples (as separate donor measurements had to be made for every experimental day).
(EPS)

S6 Fig Collected time course data for time domain fluorescent lifetime images. **A.** Multiphoton time domain time course intensity data for all donors and donor-acceptor pairs. Error bars represent the standard deviation in the normalised intensity and lifetime measurements (each individual cells time course normalised to the mean t = 0 value for that experimental day.) 90 > n < 155 for FRET Pairs and 130 < n < 450 for donor alone samples (as separate donor measurements had to be made for every experimental day). **B.** Multiphoton time domain time course lifetime data for all donors and donor-acceptor pairs. Error bars represent the standard deviation in the normalised intensity and lifetime measurements (each individual cells time course normalised to the mean t = 0 value for that experimental day.) 105 > n < 145 for FRET Pairs and 130 < n < 450 for donor alone samples (as separate donor measurements had to be made for every experimental day).
(EPS)

S7 Fig Changes in Z-Factor and FRET efficiency between single time point and repeated measurement experiments. Throughout this figure the donor involved is denoted by the shape of the point (EGFP = circle, Clv = square, mTFP = triangle and mTq2 = diamond) and the acceptor is denoted by the fill-colour of the symbol (mCh = red, mR2 = blue, YPet = yellow, Ven = green and sRCh = black) **A.** Plot of the mean Z’ of the frequency domain lifetime measurements over a 10 minute time course in 3 separate experiments against their mean FRET Efficiency for those experiments, with equivalent ‘ghost’ data-points for the single time point experiments. Pairs that are stable over time have closely overlaying solid and ghost points (*e*.*g*. Clv-mCh and EGFP-mCh.) Error bars on both axes represent standard deviation. **B.** Plot of the mean Z’ of the multiphoton time domain lifetime measurements over a 10 minute time course in 3 separate experiments against their mean FRET Efficiency for those experiments, with equivalent ‘ghost’ data-points for the single time point experiments. Pairs that are stable over time have closely overlaying solid and ghost points (*e*.*g*. Clv-mCh and EGFP-mCh.) Error bars on both axes represent standard deviation. **C.** Direct comparison of the 10 minute time course Z’ values from the two different FLIM systems with equivalent ‘ghost’ data-points for the single time point experiments; pairs that respond similarly on both systems will fall on the x = y diagonal. Pairs that are stable over time have closely overlaying solid and ghost points (*e*.*g*. Clv-mCh and EGFP-mCh.) Error bars represent standard deviation.
(EPS)

S1 Table Expected lifetime measurements of donors in the absence and presence of a FRET partner accepting energy at an efficiency of 30%, showing a greater shift in measured lifetime (τ_*D*_ *-*τ_*FRET*_) for longer lifetime donors. (DOCX)

S2 Table Frequency domain time course linear fit data. (DOCX)

S3 Table Multiphoton time domain time course linear fit data. (DOCX)

S4 Table Fluorescent donor expression plasmids. (DOCX)

S5 Table PCR primer sequences and templates. (DOCX)
