# Supplementary material for: Accepting from the best donor; analysis of long-lifetime donor fluorescent protein pairings to optimise dynamic FLIM-based FRET experiments
Source: PLoS One. 2018 Jan 2;13(1):e0183585. doi: 10.1371/journal.pone.0183585 (PMC5749721; doi:10.1371/journal.pone.0183585)
Supplement: S1 Table — (DOCX) [file pone.0183585.s008.docx]

| ***Donor*** | ***τ_D_ (ns)*** | ***τ_FRET_(ns)*** | ***τ_D_ -τ_FRET_(ns)*** |
| --- | --- | --- | --- |
| **Clv** | 3.2 | 2.2 | 1.0 |
| **EGFP** | 2.4 | 1.7 | 0.7 |
| **mTFP** | 2.8 | 2.0 | 0.8 |
| **mTq2** | 4 | 2.8 | 1.2 |

**Table S1 - Expected lifetime measurements of donors in the absence and presence of a FRET partner accepting energy at an efficiency of 30%, showing a greater shift in measured lifetime (*τ_D_ -τ_FRET_*) for longer lifetime donors.**
