# Supplementary material for: Accepting from the best donor; analysis of long-lifetime donor fluorescent protein pairings to optimise dynamic FLIM-based FRET experiments
Source: PLoS One. 2018 Jan 2;13(1):e0183585. doi: 10.1371/journal.pone.0183585 (PMC5749721; doi:10.1371/journal.pone.0183585)
Supplement: S2 Table — (DOCX) [file pone.0183585.s009.docx]

|  | **Photostability** | | | | **Lifetime Stability** | | | |
| --- | --- | --- | --- | --- | --- | --- | --- | --- |
|  | **m** | **m/m_donor_** | **R² =** | **mean% error** | **m** | **m/m_donor_** | **R² =** | **mean% error** |
| **GFP** | 0.0015 | 1.00 | 0.7749 | 32.88% | 0.0005 | 1.00 | 0.8086 | 1.37% |
| **GFP-mCh** | 0.0014 | 0.93 | 0.5234 | 34.61% | 0.0008 | 1.60 | 0.916 | 3.37% |
| **GFP-mR2** | 0.0025 | 1.67 | 0.6097 | 34.20% | 0.0009 | 1.80 | 0.9292 | 4.52% |
| **Clv** | -0.0019 | 1.00 | 0.9105 | 35.15% | 0.0002 | 1.00 | 0.3617 | 1.63% |
| **Clv-mCh** | -0.0053 | 2.79 | 0.9906 | 33.79% | 0.0003 | 1.50 | 0.6128 | 3.51% |
| **Clv-mR2** | -0.0031 | 1.63 | 0.8061 | 36.38% | 0.0001 | 0.40 | 0.0236 | 7.37% |
| **mTFP** | -0.0069 | 1.00 | 0.9822 | 34.71% | 0.0004 | 1.00 | 0.4796 | 1.92% |
| **mTFP1-Ypet** | 0.0066 | 0.96 | 0.927 | 33.71% | 0.0092 | 23.00 | 0.9947 | 3.52% |
| **mTFP1-Ven** | 0.0042 | 0.61 | 0.9917 | 27.94% | 0.0055 | 13.75 | 0.9953 | 4.43% |
| **mTFP1-sRCh** | -0.0042 | 0.61 | 0.8175 | 35.26% | 0.0012 | 3.00 | 0.8257 | 2.72% |
| **mTq2** | -0.0010 | 1.00 | 0.7944 | 35.43% | 0.0012 | 1.00 | 0.8486 | 1.88% |
| **mTq2-Ypet** | 0.0277 | 27.70 | 0.9965 | 38.55% | 0.0135 | 11.25 | 0.9937 | 11.99% |
| **mTq2-Ven** | 0.0039 | 3.90 | 0.6697 | 39.25% | 0.0047 | 3.92 | 0.9932 | 5.28% |
| **mTq2-sRCh** | 0.0014 | 1.40 | 0.9404 | 34.86% | 0.0012 | 1.00 | 0.9469 | 2.45% |

**Table S2 – Frequency Domain Time Course Linear Fit Data**
