# Supplementary material for: Accepting from the best donor; analysis of long-lifetime donor fluorescent protein pairings to optimise dynamic FLIM-based FRET experiments
Source: PLoS One. 2018 Jan 2;13(1):e0183585. doi: 10.1371/journal.pone.0183585 (PMC5749721; doi:10.1371/journal.pone.0183585)
Supplement: S3 Table — (DOCX) [file pone.0183585.s010.docx]

|  | **Photostability** | | | | **Lifetime Stability** | | | |
| --- | --- | --- | --- | --- | --- | --- | --- | --- |
|  | **m** | **m/m_donor_** | **m** | **m/m_donor_** | **m** | **m/m_donor_** | **m** | **m/m_donor_** |
| **GFP** | -0.0071 | 1.00 | 0.969 | 66.03% | 1.00E-05 | 1.00 | 0.0037 | 2.84% |
| **GFP-mCh** | -0.0167 | 2.35 | 0.968 | 55.81% | 0.0003 | 30.00 | 0.512 | 3.56% |
| **GFP-mR2** | -0.0093 | 1.31 | 0.991 | 75.07% | 0.0002 | 20.00 | 0.459 | 4.25% |
| **Clv** | -0.0158 | 1.00 | 0.9191 | 57.27% | 0.0001 | 1.00 | 0.184 | 2.93% |
| **Clv-mCh** | -0.0105 | 0.66 | 0.984 | 105.60% | 0.0002 | 2.00 | 0.219 | 3.66% |
| **Clv-mR2** | -0.0002 | 0.01 | 0.007 | 96.38% | 0.0007 | 7.00 | 0.813 | 5.67% |
| **mTFP** | -0.0177 | 1.00 | 0.935 | 83.74% | 0.0012 | 1.00 | 0.82 | 3.71% |
| **mTFP1-Ypet** | -0.0267 | 1.51 | 0.978 | 45.32% | 0.0007 | 0.58 | 0.659 | 10.76% |
| **mTFP1-Ven** | -0.0049 | 0.28 | 0.558 | 64.93% | 0.0002 | 0.17 | 0.086 | 4.70% |
| **mTFP1-sRCh** | -0.004 | 0.23 | 0.733 | 69.88% | -0.0002 | -0.17 | 0.069 | 6.21% |
| **mTq2** | -0.0112 | 1.00 | 0.909 | 70.26% | -0.0014 | 1.00 | 0.912 | 3.28% |
| **mTq2-Ypet** | -0.0086 | 0.77 | 0.769 | 41.75% | -0.0052 | 3.71 | 0.941 | 25.99% |
| **mTq2-Ven** | -0.0122 | 1.09 | 0.597 | 79.84% | -0.0002 | 0.14 | 0.379 | 4.13% |
| **mTq2-sRCh** | -0.0021 | 0.19 | 0.153 | 79.05% | -0.0004 | 0.29 | 0.597 | 3.39% |

**Table S3 – Multiphoton Time Domain Time Course Linear Fit Data**
