# Supplementary material for: Accepting from the best donor; analysis of long-lifetime donor fluorescent protein pairings to optimise dynamic FLIM-based FRET experiments
Source: PLoS One. 2018 Jan 2;13(1):e0183585. doi: 10.1371/journal.pone.0183585 (PMC5749721; doi:10.1371/journal.pone.0183585)
Supplement: S4 Table — (DOCX) [file pone.0183585.s011.docx]

| *Donor Fluorophore* | *Vector Backbone* | *Addgene Plasmid #* |
| --- | --- | --- |
| Clv | pcDNA3 | 40259 |
| EGFP | pEGFP (Clontech) | N/A |
| mTFP | pEGFP-based | N/A |
| mTq2 | pCMV-based | 54843 |

**Table S4 - Fluorescent donor expression plasmids**
