# Supplementary material for: Accepting from the best donor; analysis of long-lifetime donor fluorescent protein pairings to optimise dynamic FLIM-based FRET experiments
Source: PLoS One. 2018 Jan 2;13(1):e0183585. doi: 10.1371/journal.pone.0183585 (PMC5749721; doi:10.1371/journal.pone.0183585)
Supplement: S5 Table — (DOCX) [file pone.0183585.s012.docx]

| Fluorophore | Forward Primer | Reverse Primer | Original Plasmid |
| --- | --- | --- | --- |
| Clv (donor) | *TCC ACC GGC TAG CGC TAT GGT GAG CAA* | *CGG GAT CCT GAG TCC GGC CGG ACT TGT ACA GCT CGT C* | pcDNA3-Clover (Addgene #40259) |
| mTFP (donor) | *TCC ACC GGC TAG CGC TAT GGT GAG CAA* | *CGG GAT CCT GAG TCC GGC CGG ACT TGT ACA GCT CGT C* | EKAR2G-mTFP-wt-Venus-157 (Addgene #39814) |
| mTq2 (donor) | *TCC ACC GGC TAG CGC TAT GGT GAG CAA* | *CGG GAT CCT GAG TCC GGC CGG ACT TGT ACA GCT CGT C* | pCMV-based mTq2 vector (Addgene #54843) |
| mCh (acceptor) | *GTA CGG ATC CAC CGG TCG CCA CCA TGG TGA GCA AGG GCG AG* | *GTA CTC TAG AGT CGC GGC CGC TTT ACT TGT ACA GCT CGT C* | pmCherry (Clontech) |
| mR2 (acceptor) | *GTA CGG ATC CAC CGG TCG CCA CC****A TG****G TGT CTA AGG GC GAA* | *GTA CTC TAG AGT CGC GGC CGC TTT ACT TGT ACA GCT CGT C* | pcDNA3-mRuby2 (Addgene #40260) |
| sRCh  (acceptor) | *GTA CGG ATC CAC CGG TCG CCA CCA TGG TGA GCA AGG GCG AG* | *GTA CTC TAG AGT CGC GGC CGC TTT AAG CTC GAG ATC TGA* | pmGFP-sReACh (Addgene # 21947) |
| Ven (acceptor) | *GTA CGG ATC CAC CGG TCG CCA CCA TGG TGA GCA AGG GCG AG* | *GTA CTC TAG AGT CGC GGC CGC TTT ACT TGT ACA GCT CGT C* | EKAR2G-mTFP-159-Venus-wt (Addgene #39823) |
| YPet (acceptor) | *ATT AGG ATC CAC CGG TCG CCA CCA TGT CTA AAG GTG AAG AA* | *ATT ATC TAG AGT CGC GGC CGC TTT AGT GGT GGT GGT G* | ECFP-Ypet Src Biosensor, described in [[32](#_ENREF_32)] |

**Table S5 – PCR primer sequences and templates**
